# *Plasmodium falciparum* *var* Gene Is Activated by Its Antisense Long Noncoding RNA

**DOI:** 10.3389/fmicb.2018.03117

**Published:** 2018-12-18

**Authors:** Qingqing Jing, Long Cao, Liangliang Zhang, Xiu Cheng, Nicolas Gilbert, Xueyu Dai, Maoxin Sun, Shaohui Liang, Lubin Jiang

**Affiliations:** ^1^Unit of Human Parasite Molecular and Cell Biology, Key Laboratory of Molecular Virology and Immunology, Institut Pasteur of Shanghai, Chinese Academy of Sciences, Shanghai, China; ^2^University of Chinese Academy of Sciences, Beijing, China; ^3^Clinical Laboratory Medicine, Changzhi People’s Hospital, Changzhi, China; ^4^Department of Parasitology, School of Basic Medical Science, Wenzhou Medical University, Wenzhou, China; ^5^Institut de Médecine Régénératrice et de Biothérapie, INSERM U1183, CHU Montpellier, Montpellier, France; ^6^ShanghaiTech University, Shanghai, China

**Keywords:** malaria, *Plasmodium falciparum*, *var* gene, long non-coding RNA, T7 RNA polymerase

## Abstract

*Plasmodium falciparum* erythrocyte membrane protein 1, encoded by *var* gene, is an immunodominant antigen mediating immune evasion in humans. At a given time, only a single *var* gene is commonly expressed in one parasite. However, the regulation mechanism of *var* transcription remains largely unknown. In this study, we identified the antisense long non-coding RNA (aslncRNA) derived from *var* intron as an activation factor for the corresponding *var* gene. The exogenous artificial *var* aslncRNA transcribed by T7 RNA polymerase from episome can specifically activate the homologous *var* gene, and the exogenous aslncRNA activates transcription of both *var* mRNA and endogenous aslncRNA in a manner independent of the conserved intron sequence within the *var* gene family. Interestingly, the newly activated *var* gene and the previously dominant *var* gene then could be co-expressed in the same parasite nuclei, which suggests that the aslncRNA-mediated *var* gene activation could escape from the control of mutually exclusively expression of the *var* gene family. Together, our work shows that *var* aslncRNA is the activator responsible for *var* gene transcriptional regulation.

## Introduction

*Plasmodium falciparum*, one pathogen of malaria, caused more than 200 million infections and 445,000 deaths in 2016 (WHO, 2017). It parasitizes and reproduces in erythrocytes of infected individuals. On the surface of infected erythrocytes, multiple proteins can be expressed and presented by the malaria parasite, such as erythrocyte membrane protein 1 (PfEMP1), Rifin, Stevor (Dzikowski et al., 2006). PfEMP1, as an immunodominant antigen of *P. falciparum* (Leech et al., 1984), encoded by *var* gene, possesses the characteristic of antigen polymorphism. This protein is attributed with parasite escape from the host immune system and allows infected erythrocytes to adhere onto uninfected erythrocytes, a process related to disease severity (Rowe et al., 1995).

In *P. falciparum* 3D7, there are about 60 *var* gene members with several similar gene features, including polymorphic exonI, conserved exonII and a bi-directional promoter-activity in the intron (Calderwood et al., 2003). It is reported that only one *var* gene is exclusively expressed at ring stage of a single parasite cell, while other *var* genes are silent, referred to mutually exclusive gene expression (MEE) (Scherf et al., 1998). This phenomenon has also been found in antigen families of other parasite species (Pays et al., 2004; Prucca et al., 2008). Two main mechanisms are widely accepted for MEE regulation of multiple gene families in many other species: DNA rearrangement and epigenetic modification (Deitsch et al., 2009). However, it has been demonstrated that, in *P. falciparum*, DNA rearrangement and RNA interference could not be the main mechanism of *var* gene expression regulation (Scherf et al., 1998; Baum et al., 2009). Many other factors have been found to contribute to the regulation of *var* gene transcription, such as *cis*-elements (Deitsch et al., 2001; Calderwood et al., 2003), *trans*-factors (Voss et al., 2003; Fraschka et al., 2016), epigenetic markers (Lopez-Rubio et al., 2007; Volz et al., 2012; Jiang et al., 2013), and higher order chromatin structure (Duraisingh et al., 2005; Ralph et al., 2005b). Nevertheless, the whole MEE mechanism of *var* gene still remains unclear.

Recently, *var* gene antisense long non-coding RNAs (aslncRNAs) have emerged as new regulating candidates for *var* gene transcription. The *var* aslncRNAs are originated from *var* intron and extended to exonI. They are retained in the nucleus and are regarded as potential regulators (Epp et al., 2009). Previous studies indicate that the transcription of a particular aslncRNA correlated with the activation of the corresponding *var* gene (Jiang et al., 2013; Avraham et al., 2015). And other evidences suggest that aslncRNA is involved in the activation of *var* gene promoter (Avraham et al., 2015). However, Ralph and collaborators state that the silencing or activation of *var* gene is not overall correlated with these antisense sterile transcripts (Ralph et al., 2005a). In addition, the intron deletion of the *var2csa*, a conserved member of *var* family, could promote its transcriptional level, which also implies that the aslncRNA is not essential for *var* gene activation (Bryant et al., 2017). Therefore, it is still a controversial issue whether the *var* aslncRNA is involved in *var* transcription regulation.

To identify the role of aslncRNA in the *var* gene activation, artificial *var* aslncRNAs in this study were generated using T7 RNA polymerase in *P. falciparum*, and the *var* expression patterns were analyzed. We found that the artificial aslncRNA could specifically induce the corresponding *var* gene transcription, and the exonI region of this sterile transcript was capable of *var* specific activation. Additionally, transcription of the artificial *var* aslncRNA also induced the aslncRNA promoter activity of the corresponding *var* intron. RNA-FISH of *var* mRNA indicated that the previously dominant and newly induced *var* gene mRNAs could exist in one parasite. These findings demonstrated that the *var* aslncRNA exerted activatory function on *var* gene transcription and provided a theoretical basis for further researches on *var* MEE regulation.

## Materials and Methods

### Parasites *in vitro* Culture and Transfection

*Plasmodium falciparum* 3D7 strain C8, G4, and *P. falciparum* NF54 strain A3 were cultured as described previously (Cranmer et al., 1997). The plasmid was transfected into the parasites by electrotransformation (Gene Pulser Xcell, BIO-RAD) as mentioned before (Rug and Maier, 2012), and 2 μg/ml blasticidin S (BSD, Invitrogen) was used for transformants selection.

### Mapping 5′ End of *var* aslncRNA

The 5′ end RACE (rapid amplification of cDNA ends) of the *PF3D7_0617400* aslncRNA was performed with SMART RACE cDNA kit (TAKARA) according to the manufacturer’s instructions. *P. falciparum* NF54 strain A3 was harvested at ring stage, and the total RNA was extracted with TRIzol (Invitrogen). Five microgram total RNA of A3 strain was prepared for 5′ end determination of *PF3D7_0617400* aslncRNA. The primer for 5′ end identification is listed in Supplementary Table S1.

### Reverse Transcription Quantitative PCR (RT-qPCR)

One microgram of the extracted total RNA of *P. falciparum* was reverse transcribed using FastQuant RT Kit (Tiangen) according to its standard manuals. The RT-qPCR was performed as described (Salanti et al., 2003), and *serine-tRNA ligase* (*PF3D7_0717700*) was used as an internal control. All the RT-qPCR primer pairs for mRNA or lncRNA detection are listed in Supplementary Table S1.

### Construction of *var* aslncRNA Expression Vectors

**FIGURE 1:**
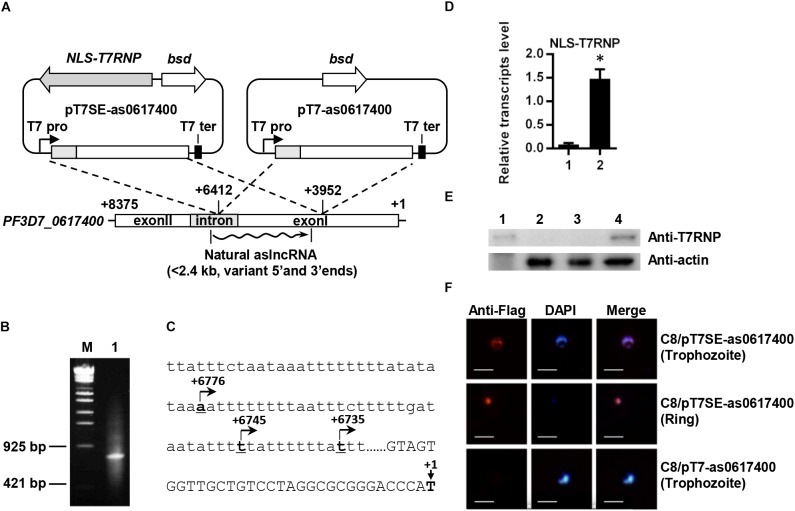
Schematic diagram of plasmids for expressing the *var* aslncRNA and 5′ end identification of *PF3D7_0617400* aslncRNA. **(A)** Schematic diagram of pT7SE-as0617400 and pT7-as0617400. The translation initial site of ATG of *PF3D7_0617400* is marked as +1 and the stop codon is marked with +8375, the ∼2.5 kb *var* fragment (from +6412 to +3952) was amplified and inserted into pT7SE and pT7, respectively. The wavy line is the natural *var* aslncRNA. T7 pro, T7 promoter; T7 ter, T7 terminator. **(B)** 5′ end identification of *PF3D7_0617400* aslncRNA. PCR products of 5′ end RACE obtained according to the standard manuals. M: the DNA marker λ-EcoT14 I digest (TAKARA), lane1: PCR products of 5′ RACE. **(C)** The *PF3D7_0617400* aslncRNA transcriptional start sites. The translation start site of *PF3D7_0617400* is marked as +1 on the schematic diagram. Capital letters are the coding sequence of the *var* exonI and the lower letters are intron sequence. The transcriptional start sites of the aslncRNAs are marked with underlined numbers and arrows. The underlined letters locations are displayed relative to translation start site, and the arrows indicate the aslncRNA transcription directions. **(D)** RT-qPCR quantification of the NLS-T7RNP transcription in C8/pT7- as0617400 (**1**) and C8/pT7SE-as0617400 (**2**). Relative transcripts numbers are normalized to *serine-tRNA ligase* gene (*PF3D7_0717700*). ^∗^*P* < 0.05 by paired two-tailed Student’s *t*-test. The RT-qPCR results are representative of three independent experiments with data indicating the mean +SD. **(E)** Western blot analysis of NLS-T7RNP expression in C8/pT7-as0617400 and C8/pT7SE-as0617400. The NLS-T7RNP is detected by anti-T7RNP antibody. Lane1: recombinant NLS-T7RNP expressed in *E. coli* BL21, Lane2: the wild type parasite 3D7 strain C8, Lane3: C8/pT7-as0617400, Lane4: C8/pT7SE-as0617400. **(F)** The subcellular localization of NLS-T7RNP in the transformant C8/pT7SE-as0617400 and C8/pT7-as0617400. NLS-T7RNP is marked with anti-Flag antibody (red) and is the nuclear marked with DAPI (blue). The scale bar is 25 μm.

To construct pCC4-NLS-T7RNP, the coding sequence of T7 RNA polymerase was amplified from *Escherichia coli* BL21(DE3) genome and inserted between XhoI and SmaI sites of pCC4 (Maier et al., 2006). The nucleus localization signal sequence of yeast *Gal4p* (1–222 bp) from pGKBT7 (Clonetech) and Flag-tag were fused on the N-terminal of T7 RNA polymerase. This fusion protein was named NLS-T7RNP. T7 promoter and terminator were amplified by overlapping PCR and cloned into pCC4-NLS-T7RNP at the AvrII site, which was named “pT7SE.” According to the description of *PF3D7_0617400* aslncRNA in (Epp et al., 2009), different artificial *PF3D7_0617400* aslncRNA templates, almost covering natural aslncRNA, were, respectively, inserted in SmaI restriction site downstream of T7 promoter (Figure 1A). In these plasmids, the *bsd* gene can be expressed in the *P. falciparum* and confer resistance of BSD. Plasmids were constructed by In-Fusion technology (Vazyme). All primers used for plasmids construction are listed in Supplementary Table S1. The transcriptional activity of NLS-T7RNP was confirmed by additional experiments in *E. coli* (Supplementary Figure S1).

### The Detection of NLS-T7RNP Activity

To generate plasmid pUC15A, the replicon of pUC19 was replaced with the replicon p15A from pACYCDuet-1 (Novagen). Then the NLS-T7RNP gene was amplified from pCC4-NLS-T7RNP and inserted between HindIII and SphI sites of pUC15A, which named pUC15A-NLS-T7RNP. For the green fluorescent protein (GFP) expression, all of the *E. coli* containing plasmids were induced with 0.5 mM IPTG and cultured at 30°C for another 6 h after reaching OD_600_ of 0.8. Then GFP was detected by SDS-PAGE and Western blot. All the primers are listed in Supplementary Table S1.

### Western Blot Analysis

The parasite samples were released from erythrocytes with 0.1% saponin, resuspended with 1% SDS/1 × PBS and sonicated for 5 min on high-level power by Bioruptor UCD-200 (Diagenode). After centrifuged at 1,3000 rpm for 2 min, the supernatant samples were separated by SDS-PAGE, and then were transferred to Immobilon-P transfer membranes (Millipore). Then each membrane of the samples were blocked with 5%BSA/1 × TBST (20 mM Tris-HCl, 0.9%NaCl, 0.1%Tween-20, 5%BSA, pH 7.5) and, respectively, incubated with anti-T7RNP (Novagen) and anti-Actin antibodies (Abmart). After being washed and incubated with anti-mouse IgG conjugated with HRP (Jackson ImmunoResearch Laboratories), the signal was developed with Immobilon Western Chemiluminescent HRP Substrate (Millipore) and imaged with Tanon 6200 System.

### Immunofluorescence Assay

The immunofluorescence assay was performed to detect the subcellular localization of NLS-T7RNP in *P. falciparum* as described previously (Flueck et al., 2009). Parasites were harvested, resuspended with 1 × PBS, fixed by 4% paraformaldehyde (Electron Microscopy Sciences) at room temperature for 10 min and washed by 1 × PBS. Prepared samples were incubated with the primary antibody anti-Flag monoclonal antibody (1:1000, Abmart) and next with the secondary antibody anti-mouse IgG conjugated with DyLight 550 (1:2000, Thermo), and finally observed by Olympus IX73.

### Localization of Induced and Dominant *var* mRNAs

The DNA templates of hybridization probes for RNA fluorescent *in situ* hybridization (RNA-FISH) were generated by PCR (Supplementary Table S1). The FISH probes of *PF3D7_1240600* and *PF3D7_0617400* mRNAs were, respectively, generated and labeled with biotin or fluorescein (Biotin-High Prime and Fluorescein-High Prime kits, Roche) according to each standard manual.

For detection of *var* gene mRNA localization, FISH was carried out as reported with some modifications (Epp et al., 2009). Fixed parasites for RNA-FISH were prepared as described above in the immunofluorescence assay and placed on polylysine-coated adhesion microscope slides (Citoglas). After permeabilization with 0.1% TritonX-100/1 × PBS for 7 min, washing with 1 × PBS and blocking with 1%BSA/1 × PBS for 30 min, FISH was performed in hybridization buffer [50% deionized formamide (Ambion), 10% dextran sulfate (MW > 500,000, Sigma-Aldrich), 2 × SSPE (Ambion), 250 μg/ml sheared salmon sperm DNA] with 70 ng/μl of each labeled probe at 37°C overnight. Slides were then washed with 50%formamide/2 × SSC and 2 × SSC (Ambion), blocked with 5%BSA/2 × SSC for 30 min, incubated with streptavidin-Alexa Fluor 594 (Thermo) for 30 min and washed with 2 × SSC. Finally, the results of RNA-FISH for *PF3D7_1240600* and *PF3D7_0617400* mRNAs were recorded by Olympus FV-1200.

## Results

### 5′ End Identification of the *var* Gene aslncRNA

Published data demonstrated a positive correlation between active *var* gene and its aslncRNA (Jiang et al., 2013; Avraham et al., 2015), which was also repeated in this study. The dominant expressed *var* gene was identified as *PF3D7_0617400* by RT-qPCR in *P. falciparum* NF54 strain A3 (Supplementary Figure S2A). The *PF3D7_0617400* aslncRNA was also dominantly expressed among the selected *var* antisense transcripts in A3 strain (Supplementary Figure S2B), therefore, this strain was used for mapping 5′ end of the *PF3D7_0617400* aslncRNA. To do so, total RNA of A3 strain was used, and a PCR fragment of ∼800 bp was obtained for sequencing (Figure 1B). Three transcriptional start sites of the *PF3D7_0617400* aslncRNA were identified and mapped in the intron thymine-rich region, locating at 6776, 6745, and 6735 bp downstream of the translation start site (Figure 1C).

### The *var* Gene Could Be Activated by Its aslncRNA

To identify the function of *var* aslncRNA, its template was cloned into the plasmids pT7SE and pT7. Because of the very high thymine content (73.9% in the first 176 bp) and short repeats at the 5′ end of *PF3D7_0617400* aslncRNA (Figure 1C and Supplementary Figure S3), the first 364 bp of the *PF3D7_0617400* aslncRNA template was unable to be obtained by PCR, so the artificial *var* aslncRNA was started from 6412 bp downstream of the *PF3D7_0617400* start codon (Figure 1A).

The *P. falciparum* 3D7 strain C8 was, respectively, transfected with pT7-as0617400 or pT7SE-as0617400 and selected by BSD. As shown in the RT-qPCR results and western blot analysis, NLS-T7RNP was successfully transcribed and expressed in C8/pT7SE-as0617400 but not C8/pT7-as0617400 (Figures 1D,E). Furthermore, in the case of C8/pT7SE-as0617400, we expected to detect co-localization of the NLS-T7RNP with the nuclear DAPI staining in ring-stage and trophozoite-stage, which was also confirmed by immunofluorescence assay (Figure 1F). It indicated the proper and efficient transport of the protein into nucleus with the nuclear localization signal of Gal4p (Wittayacom et al., 2010), which helps NLS-T7RNP to function in the nucleus.

**FIGURE 2:**
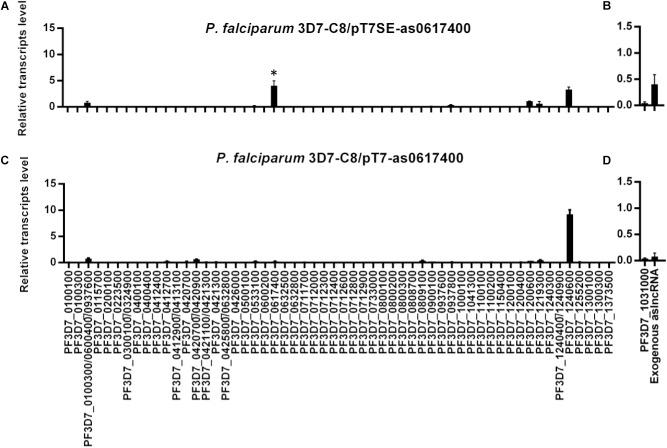
The activation of silent *var* gene by its artificial aslncRNA. The expression of *var* family and artificial aslncRNA were detected in the transformant C8/pT7SE-as0617400 (**A,B**, respectively) and the negative control C8/pT7SE-as0617400 (**C,D**, respectively). *PF3D7_0617400* was distinct expressed in C8/pT7SE-as0617400 but not C8/pT7-as0617400. ^∗^*P* < 0.05 by paired two-tailed Student’s *t*-test. **(A–D)** Relative transcripts numbers are normalized to *serine-tRNA ligase* gene (*PF3D7_0717700*). The RT-qPCR results are representative of three independent experiments with data indicating the mean+SD.

Next, total RNA of the tightly synchronized parasites C8/pT7SE-as0617400 and C8/pT7-as0617400 was harvested at 10–16 h post invasion and reverse transcribed into cDNA. The exogenous *PF3D7_0617400* aslncRNA from episome in the transformants were detected by the RT-qPCR with specific primers p1/p2 (Supplementary Table S1). The gene *PF3D7_1031000* (previously named *pfs25*), silenced in asexual blood stage, was chosen as a negative control (Dechering et al., 1999). Compared with *PF3D7_1031000*, the exogenous aslncRNA was transcribed successfully in C8/pT7SE-as0617400 but not in C8/pT7-as0617400 (Figures 2B,D), due to the lack of T7 RNA polymerase activity in C8/pT7-as0617400. Then the expression pattern of the *var* family was measured in both transformants. *PF3D7_1240600*, the dominant-expressed *var* in the wild type C8 strain (Supplementary Figure S2A), was still expressed in the two transformants (Figures 2A,C). Nevertheless, unlike the wild type and the negative control strain C8/pT7-as0617400, another *var* gene *PF3D7_0617400* mRNA was found to be transcribed at a high level in C8/pT7SE-as0617400 (*P* < 0.05, Figure 2A). It seemed that the expression of the *PF3D7_0617400* aslncRNA resulted in the activation of the corresponding silenced *var* gene.

To confirm that the expression of *PF3D7_0617400* was specifically activated by aslncRNA but not switched on in a long-term culture, pT7SE-as0617400 was transfected into G4 strain whose dominant *var* gene is *PF3D7_0711700* (Supplementary Figure S2A). Similarly, *PF3D7_0617400* was also detected to be active besides the dominant *var PF3D7_0711700* (Supplementary Figure S4). These results indicated that the *var* gene aslncRNA could induce the corresponding *var* gene transcription.

### Conserved TG Motif of *var* aslncRNA Is Not Essential for aslncRNA-Mediated *var* Activation

Previous studies revealed that the TG motifs were present in almost all the *var* gene introns and involved in *var* gene expression regulation (Avraham et al., 2012). The conserved TG motifs were also found in the intron region of *PF3D7_0617400* aslncRNA (Supplementary Figure S3) but not in the exonI region of the artificial aslncRNA.

**FIGURE 3:**
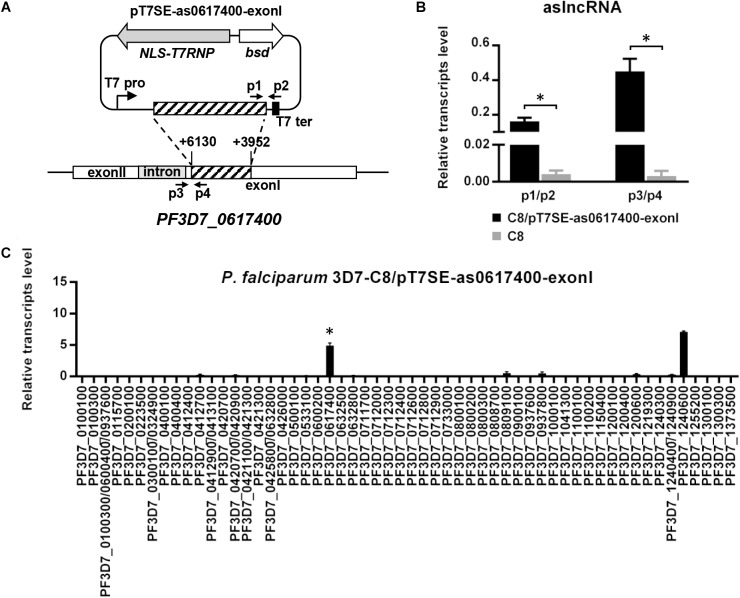
Silent *var* gene could be activated by its artificial aslncRNA lacking the intron sequence. **(A)** The schematic diagram of the episome for expressing the artificial *PF3D7_0617400* aslncRNA lacking intron sequence. This plasmid was then used to transfected into C8 strain. Specific primer pairs p1/p2 and p3/p4 were used for the exogenous and endogenous artificial aslncRNA detection, respectively. T7 pro, T7 promoter; T7 ter, T7 terminator. **(B)** The expression of the exogenous and endogenous *PF3D7_0617400* aslncRNAs in C8/pT7SE-as0617400-exonI (black) and C8 strain (gray). **(C)** The *var* gene expression pattern in C8/pT7SE-as0617400-exonI. In this transformant, the artificial *PF3D7_0617400* aslncRNA was lacking intron region. *PF3D7_0617400* was distinct expressed in C8/pT7SE-as0617400-exonI but not C8/pT7-as0617400. (**B,C**, respectively) Relative transcripts numbers are normalized to *serine-tRNA ligase* gene (*PF3D7_0717700*). The RT-qPCR results are representative of three independent experiments with data indicating the mean+SD. ^∗^*P* < 0.05 by paired two-tailed Student’s *t*-test.

To identify whether the conserved TG motif was critical to aslncRNA-mediated *var* activation, the artificial *PF3D7_0617400* aslncRNA template without the intron sequence was designed (Figure 3A) and expressed in the C8 strain. Then the transformant C8/pT7SE-as0617400-exonI was harvested at ring stage. As indicated in Figure 3B, the transcription of the artificial *PF3D7_0617400* aslncRNA lacking TG motifs was detected in this transformant, and the aslncRNA also activated the corresponding *var* gene transcription at a high level compared with C8/pT7-as0617400 (*P* < 0.05, Figure 3C). The result showed that aslncRNA lacking the conserved motifs still retained the function of *var* activation. So that, the TG motif was not the essential element for *var* gene activation.

### The *var* aslncRNA Promoter in Intron Is Also Activated in the Induced *var* Gene

Some studies reported that the *var* gene and the corresponding aslncRNA were co-expressed (Jiang et al., 2013; Avraham et al., 2015). Thus, the exogenous aslncRNA activated the corresponding *var* gene, we decided to check if the aslncRNA promoter in the corresponding intron was also activated.

In order to test this, specific primer pairs for RT-PCR were prepared to distinguish the exogenous (p1/p2) and endogenous (p3/p4) transcripts in C8/pT7SE-as0617400-exonI (Figure 3A). The RT-qPCR data revealed the distinct transcription level of endogenous *PF3D7_0617400* aslncRNA, whereas, no detectable expression of endogenous *PF3D7_0617400* aslncRNA was found in the wild type C8 strain at the ring stage (Figure 3B). These results indicated that the *PF3D7_0617400* intronic promoter was active to transcribe antisense transcripts when this *var* was induced by its corresponding artificial aslncRNA, and it further confirmed the co-activation of the promoters from the *var* gene and its intron. Regarding of the bi-directional promoter activities in the *var* gene intron, the sense lncRNA derived from the *PF3D7_0617400* intron was also measured with specific RT-qPCR primers. However, a very low level of the sense lncRNA was detected in C8 strain at the same stage, suggesting the intronic promoter responsible for the sense lncRNA synthesis remained silent at the ring stage (Data not shown).

### Dominant and Induced *var* Genes Are Transcribed in Single Parasite

The RT-qPCR data indicated that both of the dominant and induced *var* genes could be detected in the transformant population (Figures 2A, 3C, and Supplementary Figure S4A). We were then further checked whether the induced *var* mRNA was exclusively expressed or simultaneous expressed with the dominant *var* gene in single parasite.

**FIGURE 4:**
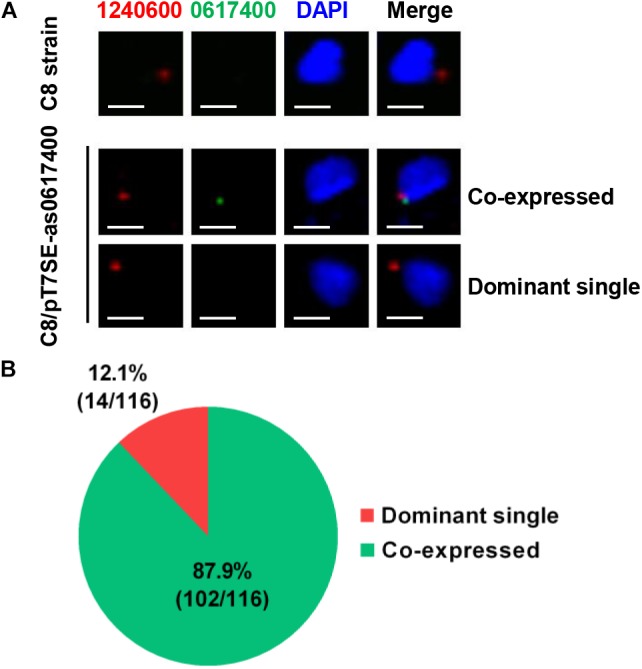
The subcellular location of *var* gene mRNAs by RNA-FISH. **(A)** The fluorescent microscopy images of the dominant and induced *var* mRNAs in the wild type C8 strain and the transformant C8/pT7SE- as0617400. The parasites were harvested at ring stage. The subcellular localization of the dominant *PF3D7_1240600* (red) and induced *var* *PF3D7_0617400* (green) were detected by RNA-FISH, the nucleus was stained with DAPI (blue). The scale bar is 2 μm. **(B)** In 116 parasites of C8/pT7SE-as0617400, *PF3D7_0617400* mRNA (green) was observed together with *PF3D7_1240600* mRNA (red) in 102 parasites, and only 14 parasites predominantly transcribed *PF3D7_1240600*.

Thus, RNA-FISH probes were prepared and applied to detect dominant and induced *var* mRNAs. In the wild type C8 strain, according to its *var* expression pattern, *PF3D7_1240600* mRNA (red) was detected while the observation of *PF3D7_0617400* mRNA (green) was failed (Figure 4A). In C8/pT7SE-as0617400, the *PF3D7_0617400* mRNA (green) could be observed together with *PF3D7_1240600* mRNA (red) in most parasite (102/116), and only 14 out of 116 parasites predominantly transcribed *PF3D7_1240600* (Figures 4A,B). No parasite was found with a single green signal in C8/pT7SE-as0617400. This observation revealed the widespread co-expression of two *var* genes in one parasite. It suggested that the induced *var* gene by exogenous aslncRNA could get outside the MEE mechanism control and co-transcribed with previously dominant *var* gene in one cell.

In addition, it was noteworthy that the dominant *PF3D7_1240600* expression level in C8/pT7SE-as0617400 was only half of that in the negative control (Figures 2A,C), which was probably caused by competitive transcription between the induced and dominant *var* genes in one parasite.

## Discussion

It was described previously that the *var* aslncRNA co-expressed with its *var* gene (Jiang et al., 2013; Avraham et al., 2015). Nevertheless, it is disputable whether aslncRNA is involved in *var* transcription regulation. Therefore, the role of aslncRNA in *var* gene regulation is significant to be identified, which also will help to understand the *var* gene MEE mechanism. Remarkably, the *var* intron, as promoter of the *var* aslncRNA, was regarded to be involved in *var* expression regulation by potential *cis–*elements interactions with other factors (Calderwood et al., 2003; Voss et al., 2006; Dzikowski et al., 2007; Avraham et al., 2012). Therefore, it may disrupt functions or interactions of these factors by using genomic engineering strategy to modify the endogenous *var* gene intron. To avoid these interference, we investigated the *var* gene aslncRNA function via an episome introduced into *P. falciparum*. In such system, the genome is not modified and no additional intron sequence is introduced into the parasite, which should avoid disruptions or changes of some potential *cis*- or *trans-* elements interactions.

With T7 RNA polymerase system, the *var* aslncRNA is firstly identified as a functional activator to be responsible for the transcriptional regulation of corresponding *var* gene. In this study, the *var* gene *PF3D7_0617400* is activated following the transcription of the additional aslncRNA from episome, showing that aslncRNA is a key activator on *var* gene transcription. Interestingly, *PF3D7_0712600*, which possess 73% identity with the exonI region of the *PF3D7_0617400* aslncRNA template, failed to be active in all the transformants of C8/pT7SE-as0617400, C8/pT7SE-as0617400-exonI, and G4/pT7SE-as0617400 according to the RT-qPCR results (Figures 2A, 3C, and Supplementary Figure S4A). The data indicate that aslncRNA-mediated *var* activation is specific.

The result of RNA-FISH reveals that the previously dominant and newly induced *var* could be co-expressed in one parasite, and a competitive transcription between the dominant and induced *var* is also discovered in transformants (Figures 2A, 4). The induced *var* gene competes with the dominant *var* gene for some limited factors (e.g., transcriptional factors, chromatin remodeling factors and subnuclear compartment), which results in a transcription decrease of the latter. Although this status of co-expression potentially resembles to the transition state of the *var* gene switching, we could not manage to promote further development from co-existence to exclusive transcription of induced *var* by a long-term culture (Supplementary Figure S5). This implies other factors involved in the *var* MEE maintenance and development. Thus, we are more likely to regard the aslncRNA-mediated *var* activation not as the trigger but as a relative independent intermediary step of the *var* gene MEE regulation.

Generally, the long noncoding RNA (lncRNA) function in two ways: lncRNA–microRNA interaction or lncRNA–protein interaction. Mature microRNA is about 22-nucleotide RNA molecules in cytoplasm (Lund et al., 2004). If interacted with microRNA, lncRNA also should be exported into cytoplasm. The existing evidences demonstrated that *P. falciparum* *var* aslncRNA was located in cell nucleus (Epp et al., 2009). Therefore, we do not think that the *var* aslncRNA activates the corresponding *var* gene by interacting with microRNA. It is worth noting that the conserved TG motif, in all of the *var* intron region, has been demonstrated to be involved in *var* gene regulation. This element is merely found in the intron region of the *var* aslncRNA but not exonI region. In Avraham’ model, the TG motif (named “insulator-like PE” in their study, Avraham et al., 2015) bond with some silence-related factors and maintained the promoter–intron interaction, which made the *var* gene silence. The *var* aslncRNA could incorporate into chromatin in a sequence-specific manner and compete the silence-related factors with its TG motif, which resulted in the disruption of the promoter–intron interaction, then, the *var* promoter would be active. In our study, we also confirmed that *var* aslncRNA activatory function is sequence-dependent. However, we find that the *var* aslncRNA lacking the intron sequence still has capability of *var* activation (Figure 3C), which implies that the aslncRNA-mediated *var* activation is not by the way of binding with silence-related proteins with these conserved elements on the *var* aslnRNA.

Based on our data and Avraham’s study, we try to explain the mechanism underlying aslncRNA function: not only *var* but also non-*var* gene aslncRNA can specifically activate *var* gene promoter with its polymorphic sequence. As we know, Schmitz and collaborators found the DNA methyltransferase DNMT3b could be recruited by DNA-RNA triplex, and mediate CpG methylation to repress rRNA genes (Schmitz et al., 2010). Similarly, the lncRNA *MEG3* and *HOTAIR* also can regulate genes by forming DNA-RNA triplex structure (Mondal et al., 2015; Kalwa et al., 2016). These studies revealed a novel regulation mechanism associated with lncRNA. According to these data and ours, we propose that there are potential binding domains in aslncRNA which could form the DNA-RNA triplex with the corresponding DNA. Then, the RNA-DNA triplex could further recruit factors to regulate the *var* transcription.

## Author Contributions

LJ, QJ, and SL designed the experiments. QJ, LC, LZ, XC, and MS performed experiments. QJ, LC, LZ, XC, NG, XD, MS, and LJ analyzed data. QJ, LC, LJ, NG, XD, and LJ wrote the paper. All authors read, contributed feedback to, and approved the final manuscript.

## Conflict of Interest Statement

The authors declare that the research was conducted in the absence of any commercial or financial relationships that could be construed as a potential conflict of interest.
