# Supplementary material for: Plasmodium falciparum var Gene Is Activated by Its Antisense Long Noncoding RNA
Source: Front Microbiol. 2018 Dec 18;9:3117. doi: 10.3389/fmicb.2018.03117 (PMC6305453; doi:10.3389/fmicb.2018.03117)
Supplement: Supplementary file 1 [file Image_1.pdf]

Supplementary Material

Plasmodium falciparum var gene is activated by its antisense long noncoding RNA

Qingqing Jing[1,2†]*, Long Cao[1†], Liangliang Zhang[3,4†], Xiu Cheng[1,2], Nicolas Gilbert[1,5], Xueyu Dai[1,2], Maoxin Sun[1,6], Shaohui Liang[4]* and Lubin Jiang[1,2,6]*

[1]Unit of Human Parasite Molecular and Cell Biology, Key Laboratory of Molecular Virology and Immunology, Institut Pasteur of Shanghai, Chinese Academy of Sciences, Shanghai, P. R. China
[2]University of Chinese Academy of Sciences, Beijing, P. R. China
[3]Clinical laboratory medicine, Changzhi People hospital, Changzhi, Shanxi, P. R. China
[4]Department of Parasitology, School of Basic Medical Science, Wenzhou Medical University, Wenzhou, Zhejiang, P. R. China
[5]Institut de Médecine Régénératrice et de Biothérapie, INSERM U1183, CHU Montpellier, Montpellier, France
[6]Shanghai Tech University, Shanghai, P. R. China.

* **Correspondence:**
Shaohui Liang: lsh@wmu.edu.cn
Qingqing Jing: qqjing@ips.ac.cn
Lubin Jiang: lbjiang@ips.ac.cn
† These authors have contributed equally to this work.

Supplementary Figure S1

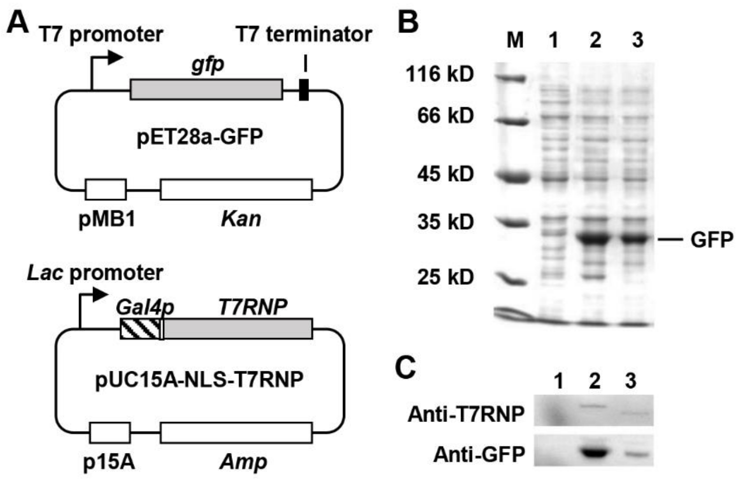

Supplementary Figure S1. The activity detection of NLS-T7RNP. (**A**) The schematic diagram of expression vectors of NLS-T7RNP and green fluorescent protein (GFP). The GFP gene is under T7

promoter control and NLS-T7RNP gene is under *lac* promoter control. The two plasmids contain different drug resistances (**kan**amycin and **amp**icillin) and replicons (pMB1 and p15A), therefore, they could be co-existed in one *Escherichia coli*. (**B**) The expression of GFP by T7RNP and NLS-T7RNP in *E. coli*. M: protein molecular weight marker. (**C**) Western blot analysis of GFP and NLS-T7RNP in *E. coli*. The T7RNP was detected with anti-T7RNP antibody and GFP was detected by anti-GFP antibody (Abmart). (**B** and **C**, respectively) 1: *E.coli* BL21, 2: *E. coli* BL21 containing pET28a-GFP and pUC15A-NLS-T7RNP, 3: *E. coli* BL21 (DE3) containing pET28a-GFP.

Supplementary Figure S2

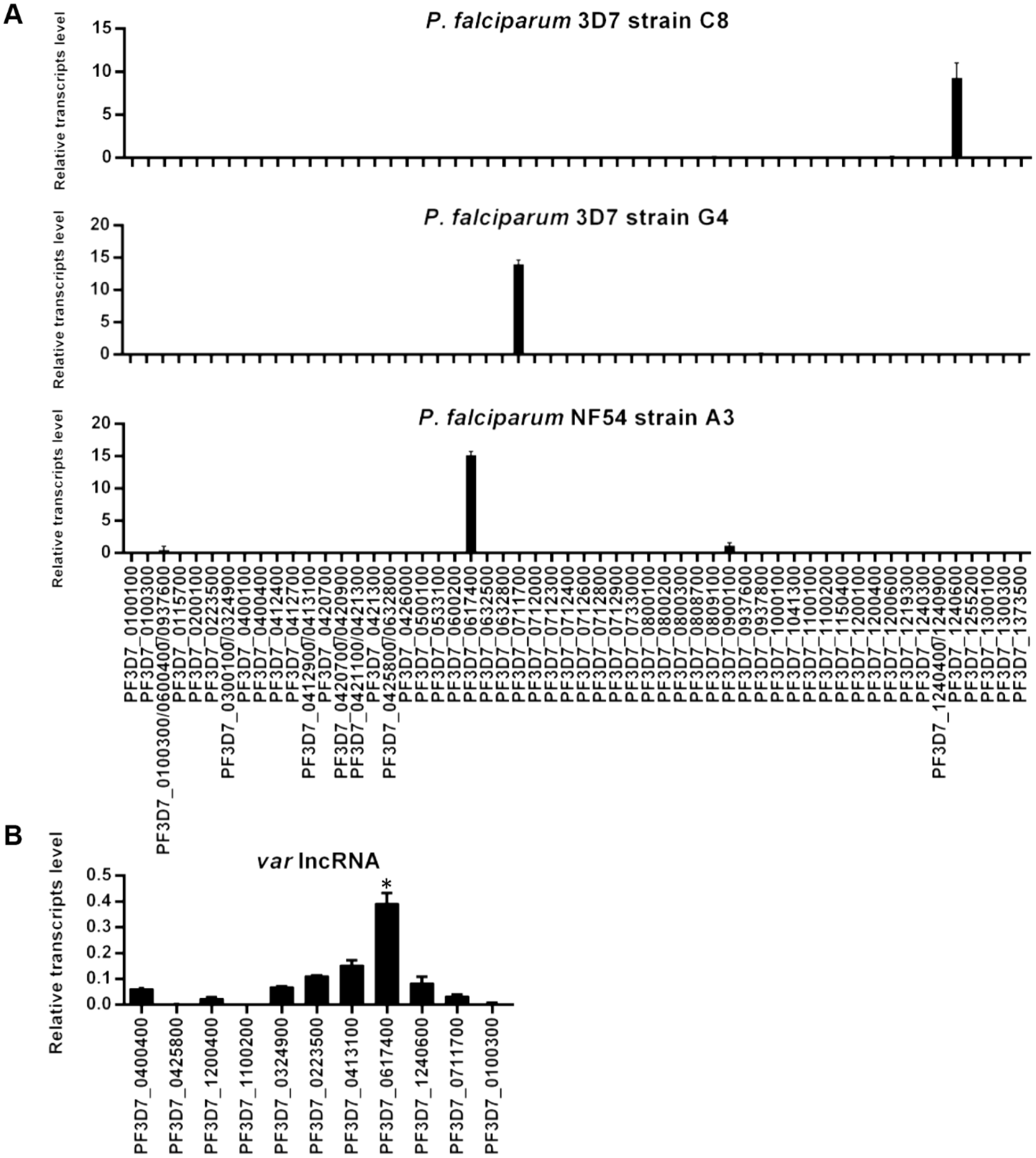

Supplementary Figure S2. The expressions of *var* genes and their aslncRNAs in different *P. falciparum* strains. (**A**) The expression patterns of *var* genes in *P. falciparum* 3D7 strain C8, G4 and NF54 strain A3. To detect the *var* expression pattern, parasites were respectively harvested at ring stage. Total RNA of each strain (C8, G4 and A3) was extracted and reverse transcribed to cDNA. The *var* expression was performed by qPCR. (**B**) The aslncRNA expression of chosen *var* genes in *P. falciparum* NF54 strain A3. Total RNA of A3 strain was extracted from ring-stage parasite, and reverse transcribed into cDNA. The relative aslncRNA level of chosen *var* genes were detected by qPCR. Compared with other chosen *var* aslncRNA, *PF3D7_0617400* was dominant expressed. **P*<0.05 by

paired two-tailed Student's *t*-test. (**A** and **B**, respectively) Relative transcripts numbers are normalized to *serine-tRNA ligase* gene (*PF3D7_0717700*). The qPCR results are representative of three independent experiments with data indicating the mean +SD. All primer pairs are listed in **Supplementary Table S1**.

Supplementary Figure S3

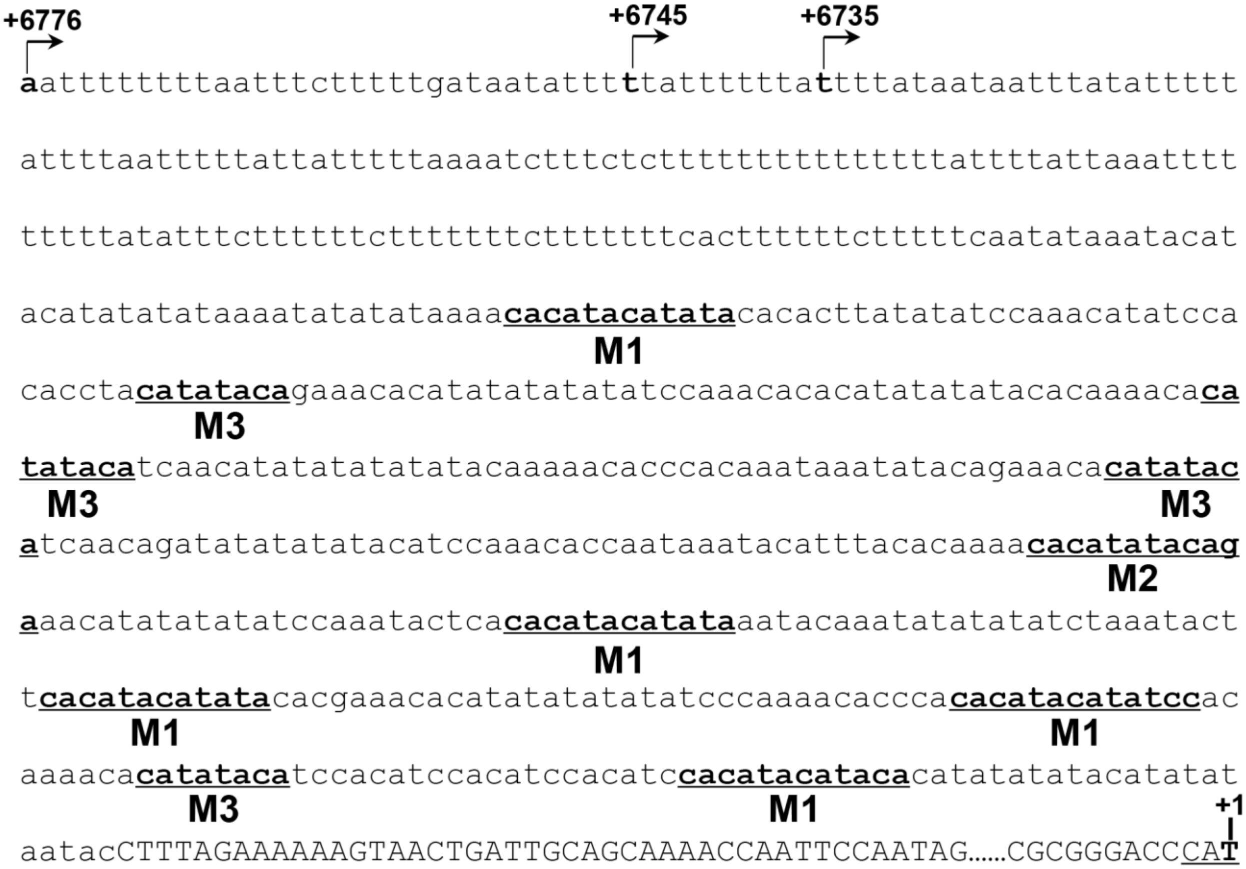

Supplementary Figure S3. The conserved TG motif in the intron region of the *PF3D7_0617400*. The TG motif contains three subtype: M1 (TGTATGTAGTG), M2 (TGTGTATATGTG) and M3 (TGTATGTG) (Avraham et al., 2012). These motifs are marked with bold letters and underline. The sequence with lower letters are the intron sequence and capital letters are the exonI region. The translation start site of *PF3D7_0617400* is marked as +1, and the letters marked with +6776, +6745 and+6735 are the aslncRNA transcriptional start sites identified in this study.

References

Avraham, I., Schreier, J., and Dzikowski, R. (2012). Insulator-like pairing elements regulate silencing and mutually exclusive expression in the malaria parasite Plasmodium falciparum. *Proc. Natl. Acad. Sci.* 109, E3678–E3686. doi: 10.1073/pnas.1214572109

Supplementary Figure S4

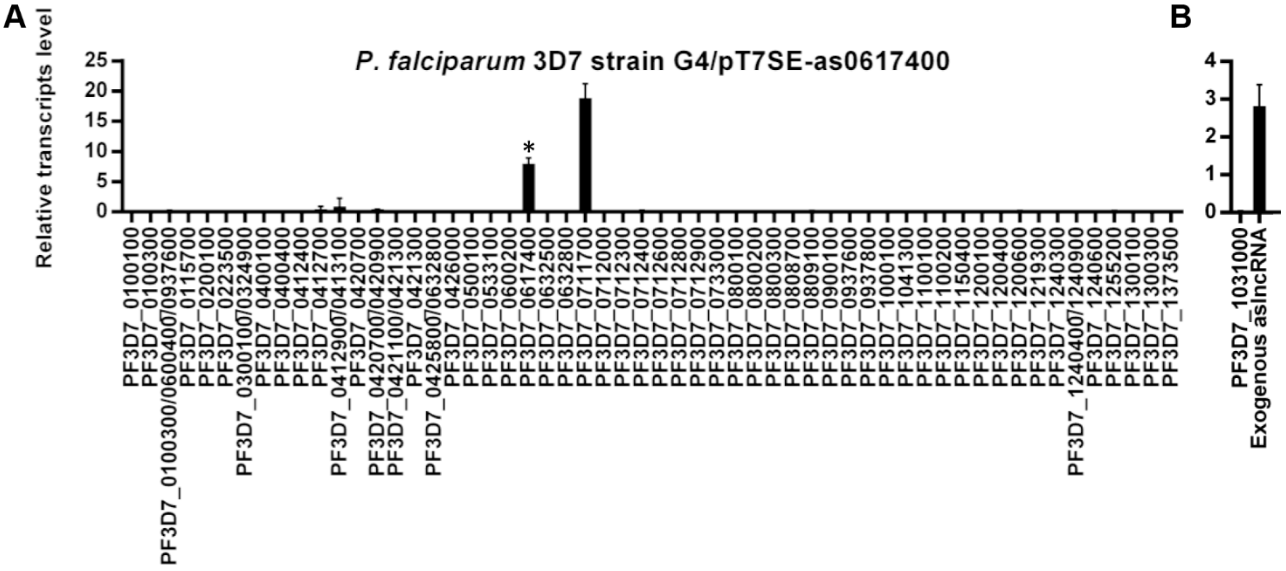

Supplementary Figure S4. The activation of silence *var* gene by its artificial aslncRNA in G4/pT7SE-as0617400. *PF3D7_0711700* is the dominant *var* in G4 strain (**Supplementary Figure S1A**). Compared with G4 strain, *PF3D7_0617400* was distinct expressed in transfected parasite G4/pT7SE-as0617400. Relative transcripts numbers are normalized to *serine-tRNA ligase* gene (*PF3D7_0717700*). The qPCR results are representative of three independent experiments with data indicating the mean +SD. **P*<0.05 by paired two-tailed Student's *t*-test.

Supplementary Figure S5

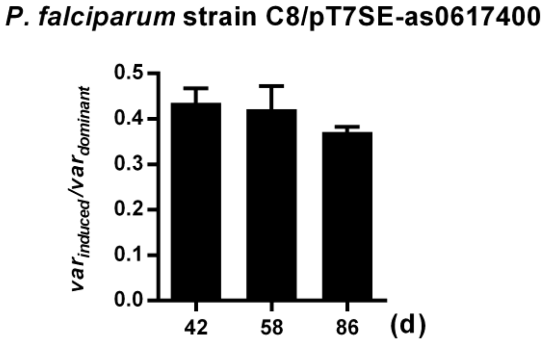

Supplementary Figure S5. The proportion of the induced *var* (*PF3D7_0617400*) and previously dominant *var* (*PF3D7_1240600*) in the transformant C8/pT7SE-as0617400. Synchronized samples were respectively harvested at 43, 58 and 86 days past electrotransformation, and *var* gene expression of each sample was detected by RT-qPCR. Relative transcripts numbers are normalized to *serine-tRNA ligase* gene (*PF3D7_0717700*). The results are representative of three independent experiments with data indicating the mean +SD.
