# Supplementary material for: Plasmodium falciparum var Gene Is Activated by Its Antisense Long Noncoding RNA
Source: Front Microbiol. 2018 Dec 18;9:3117. doi: 10.3389/fmicb.2018.03117 (PMC6305453; doi:10.3389/fmicb.2018.03117)
Supplement: Supplementary file 2 [file Table_1.pdf]

Supplementary Material

Plasmodium falciparum var gene is activated by its antisense long noncoding RNA

Qingqing Jing[1,2]†*, Long Cao[1]†, Liangliang Zhang[3,4]†, Xiu Cheng[1,2], Nicolas Gilbert[1,5], Xueyu Dai[1,2], Maoxin Sun[1,6], Shaohui Liang[4]* and Lubin Jiang[1,2,6]*

[1]Unit of Human Parasite Molecular and Cell Biology, Key Laboratory of Molecular Virology and Immunology, Institut Pasteur of Shanghai, Chinese Academy of Sciences, Shanghai, P. R. China
[2]University of Chinese Academy of Sciences, Beijing, P. R. China
[3]Clinical laboratory medicine, Changzhi People hospital, Changzhi, Shanxi, P. R. China
[4]Department of Parasitology, School of Basic Medical Science, Wenzhou Medical University, Wenzhou, Zhejiang, P. R. China
[5]Institut de Médecine Régénératrice et de Biothérapie, INSERM U1183, CHU Montpellier, Montpellier, France
[6]Shanghai Tech University, Shanghai, P. R. China.

* **Correspondence:**
Shaohui Liang: lsh@wmu.edu.cn
Qingqing Jing: qqjing@ips.ac.cn
Lubin Jiang: lbjiang@ips.ac.cn
† These authors have contributed equally to this work.

Supplementary Tables

Target	Sequence (5'-3')
**5' RACE**	
PF3D7_0617400 antisense lncRNA	GATTACGCCAAGCTTGAACCTGAAGCTGATAAAGGCCCAGTC
**Primer pairs for mRNA qPCR detection**	
var family	See reference (Salanti et al., 2003)
T7 RNA polymerase	GGAATACAAGAAGCCTAT<br>TGTTGGTGTTAATGGTAG
PF3D7_0100300	CATCATCTAAAAATCTTAATATCTA<br>CTTTATTTCATATTTATATTGTGAG
Rex1	AATCGGGTGCTCCATACAAG<br>CGTCTTTTTGTCCCTGTTCTG
**Primer pairs for *var* aslncRNA qPCR detection**	
PF3D7_0617400 aslncRNA (episome, p1/p2)	TGTGTAGAACATTTGGCGCA<br>CAAGAAAACAGATCCCCTAC
PF3D7_0617400 aslncRNA (endogenous, p3/p4)	GGAATTGGTTTTGCTGCAATC<br>CATCCACATCCACATACATAC
PF3D7_0400400	CTACTATCCCGTTTGGTATTG<br>CATCCACACGTAAACATATCC

PF3D7_0425800	TTATTTTCTACCATCCTCTGG<br>TCCAAACATACACAACATACAC
PF3D7_1200400	TTGGCATTAGGATCCATTGCT<br>AACGCTCAAACATACATATACAG
PF3D7_1100200	GCGTTGACTTACTTTTTACTC<br>CATACCCAAACATACATAAGC
PF3D7_0300100	CCGTTTGGTATTGCATTGGC<br>ACGTAAACATATCCCCCACAC
PF3D7_0223500	TGGCATTAGGATCCATTGCTT<br>TATATCCAAACACACCCACAC
PF3D7_0413100	TATTGGTTTTGCTGCGTTCAC<br>CCAAACATACCCCACAAATAC
PF3D7_1240600	ATCGGTTTTGCTGCATTCACT<br>ACAATCATATCAAACACATCCAC
PF3D7_0711700	CATCACAACCAACAACCCC<br>CACTCATACATACATATACACAC
**pT7SE and pT7 construction**	
T7 RNA polymerase	CACATTTCGAATAAACTCGAGATAACACGATTAACATCGCTAAGAAC<br>GACCTGCAGGGTACCTTACGCGAACGCGAAGT
Nuclear localization signal of Gal4p (1-222 bp)	CACATTTCGAATAAACTCGAGATGAAGCTACTGTCTTCTATCG<br>GATGTTAATCGTGTTCCCGGGTCGAGGAAAAATCAGTAGAAATAGC
Flag-tag	CTGATTTTTCCTCGAGACTACAAGGACGACGATGACAAGAACACGATTAACATC<br>GATGTTAATCGTGTTCTTGTCATCGTCGTCCTTGTAGTCTCGAGGAAAAATCAG
T7 promoter and T7 terminator (*)	Forward1:<br>CTATAGGGAGACCCGGGTTCTTGCAAGATAACTAGCATAACCCCTTGGGGCCTCT<br>Reverse1:<br>TTTCAGCAAAAAACCCCTCAAGACCCGTTTAGAGGCCCCAAGGGGTTATGCTAGTT<br>Forward2:<br>GCCAGCCTAGGAGTTCCATGGAAATTAATACGACTCACTATAGGGAGACCCGGGT<br>Reverse2:<br>TTCATATCGATAACTATCCGGATATAGTTCCTCCTTTCAGCAAAAAACCCCTCAAG
**pT7SE-as0617400 and pT7-as0617400 construction**	
PF3D7_0617400 aslncRNA template	GACTCACTATAGGGAGAGAAACACATATACATCAACAGAT<br>CAAGAAAACAGATCCCCTACTTAGCCAGTTCAGCAT
Deletion of NLS-T7RNP operon	AAGACAGATCTTCGGGCGGCCGCGAGTATTCTATAGTGTC<br>GACACTATAGAATACTCGCGGCCGCCCGAAGATCTGTCTT
**Templates for FISH probe synthesis**	
PF3D7_1240600 FISH template	ACATGACGAGGTACAGAAAG<br>GCTTGTGGTGTCACCTG
PF3D7_0617400 FISH template	CAATACTTTCCACTGATAGAGC<br>CCAAACTTCTTTTCTGTTTGCCT
**pT7SE-as0617400-exonI construction**	
PF3D7_0617400 aslncRNA template (exonI region)	ACTCACTATAGGGAGGTAACTGATTGCAGCAAAACC<br>GTTATCTTGCAAGAACCCGGGCTACTTAGCCAGTTCAGCAT
**pUC15A-NLS-T7RNP construction (**)**	
p15A replicon	CACCGCCGGACATCAGCG<br>CGGGGCATGACTAACATG

pUC19 fragment lacking replicon	GGATCTCAAGAAGATCCTTTG<br>GTGAGCTGATACCGCTCGC
NLS-T7RNP	ATGACCATGATTACGCCAAGCTTGATGAAGCTACTGTCTTCTATCG<br>GAGTCGACCTGCAGGCATTTACGCGAACGCGAAG

Supplementary Table S1. All primers used in this study. The vectors were constructed by In-Fusion technology (Vazyme). The sequence underlined is the homologous fragment for cloning as commercial standard manuals described. *: To obtain this fragment, products were amplified by two steps. The first product was amplified by primer pair Forward1/Reverse1 without template. Then, the second PCR was performed by primer pair Forward2/Reverse2 and using the first step product as template. **: p15A replicon and NLS-T7RNP were prepared by PCR. To construct pUC15A, p15A replicon was phosphorylated with T4 polynucleotide kinase, and ligated to pUC19 fragment lacking replicon with T4 DNA ligase.

References

Salanti, A., Staalsoe, T., Lavstsen, T., Jensen, A. T., Sowa, M. P., Arnot, D. E., et al. (2003). Selective upregulation of a single distinctly structured var gene in chondroitin sulphate A-adhering Plasmodium falciparum involved in pregnancy-associated malaria. *Mol. Microbiol.* 49, 179–191.
